# Case study of a world hour record simulation in an elite cyclist: Insight into task failure

**DOI:** 10.1002/ejsc.12195

**Published:** 2024-11-12

**Authors:** Mehdi Kordi, Dan Bigham, Jacob Tipper, Richard A. Ferguson, Glyn Howatson, Jonathan Wale

**Affiliations:** ^1^ Royal Dutch Cycling Federation (KNWU) Papendal Arnhem The Netherlands; ^2^ Department of Sport, Exercise and Rehabilitation Northumbria University Newcastle UK; ^3^ Ineos Grenadiers Manchester UK; ^4^ JTP Performance Coaching Birmingham UK; ^5^ School of Sport, Exercise and Health Sciences Loughborough University Loughborough UK; ^6^ Water Research Group North‐West University Potchefstroom South Africa

**Keywords:** cycling, performance, task‐success, world‐record

## Abstract

The ‘cycling hour‐record’ is one of the most prestigious events in cycling. However, little detailed analysis of such attempts is available. In preparation for a successful cycling hour‐record attempt, an elite cyclist performed a full‐hour simulation to provide insights into performance, physiological, aerodynamic and biomechanical limitations that could be identified in the preparation for a subsequent official attempt. Performance (speed, lap time, power and cadence), physiological (heart rate and estimated body temperature), aerodynamic (C_D_A, helmet angle, rotation and rock) and biomechanical (helmet, thigh and foot position changes) measurements were made throughout the attempt, in which an even‐pacing strategy was employed where the point of task failure was defined as the lap which the rider could no longer perform at the targeted lap split (16.6 s) or quicker. The cyclist did not achieve the target distance (54,000 m) during the simulation. The final distance achieved for the hour was 53,250 m. Task failure occurred at 38 min and 33 s (lap 139/34,750 m) into the simulation. Notably, there was a decrease in power output, accompanied with an increase in the estimated body temperature, changes in pedalling kinematics and an increase in aerodynamic drag. The reduction in performance (leading to task failure) during a cycling hour record simulation is underpinned by a decrease in power output as well as an increase in aerodynamic drag due to biomechanical changes in the cycling technique.

## Highlights

- An elite cyclist performed a cycling hour record simulation to provide insights into physiological, aerodynamic and biomechanical determinants of performance.
- An even‐pacing strategy was employed where the point of task failure was defined as the lap which the cyclist could no longer perform at the targeted lap split (16.6 s) or quicker.
- Task failure occurred at 38 min and 33 s (lap 139/34,750 m) into the simulation, and they failed to achieve target distance (54,000 m), achieving 53,250 m.
- The reduction in performance (leading to task failure) was underpinned by a decrease in power output as well as an increase in aerodynamic drag due to biomechanical changes in the cycling technique.

## 1 INTRODUCTION

The ‘cycling hour‐record’ is one of the most prestigious events in cycling and requires a cyclist to ride as far as possible in 1 hour from a stationary start around a velodrome track. Francesco Moser's record‐breaking mark of 51.151 km in 1984 brought in an era of rapid advances in technology, including aerodynamics (Malizia et al., 2021), which continued in the 1990s and culminated with Chris Boardman's ‘superman’ position achieving 56.375 km. This subsequently led to the international cycling governing body, Union Cycliste Internationale (UCI), banning certain positions, which resulted in a diminished number of attempts. In 2014, the UCI announced an update in the rules, allowing bicycle geometry and equipment that complies with current rules for time‐trial (TT) events to be used on the track. This permitted riders to adopt their accustomed TT positions making the cycling hour record more appealing, ushering in a Unified era (Harnish et al., 2024). At the time of writing this manuscript, a total of 26 official men's attempts have been made in this Unified era, of which 8 have succeeded in breaking the record, which currently stands at 56.792 km by Filipo Ganna.

There is no doubt that the cycling hour record represents a rare insight into the limits of human endurance performance. The basis of the task is to achieve a high average speed >55kmh (∼35mph), requiring the development of high levels of sustained power output (Malizia et al., 2021). The underpinning physiological determinants of endurance performance are well‐established as outlined by Joyner and Coyle (2008) and are crucial for task success (Joyner et al., 2008). They include highly developed aerobic capacity (VO_2max_) and submaximal thresholds (critical power (CP)/lactate threshold) permitting high levels of sustainable power. Another critical component of the endurance performance model is mechanical efficiency, which is underpinned by multiple physiological determinants as well as the biomechanics of the cycling technique.

Cycling hour record attempts that fail are usually due to the inability to achieve or maintain the required speed throughout the hour, which can be considered as one definition of task failure, the determinants of which are complex and multifactorial. Clearly, muscle fatigue, defined as the failure to maintain the required power output (Porter, 1981) and underpinned by the physiological determinants described above, will play a prominent role. However, it is likely that changes in the body position (that would influence aerodynamic properties of cycling) and biomechanics of the cycling technique are also likely to play a role but are not usually accounted for.

Given the rarity of cycling hour record attempts, using a case report methodology provides the only realistic opportunity to provide reliable observations from a real‐world scenario. Indeed, little detailed analyses of such attempts have been performed, with only one case report published in 2000. Padilla et al. reported observations in an elite cyclist from simulated efforts during the build‐up to a successful attempt that exceeded 53,000 m (Padilla et al., 2000). Despite the potential benefit of establishing a greater understanding of the overall demands of cycling hour record attempts, it is uncommon for riders who attempt the record to collect extensive data due to (1) regulations stipulating cyclists are blind to any data that is directly collected from the bicycle such as cadence, speed and power; (2) the potential aerodynamic penalty of wearing the instruments and/or (3) the potential issues setting up the instruments when athlete preparation is of greater importance.

The following case report is a retrospective analysis of a simulated cycling hour record performed by an elite‐level cyclist in preparation for an official attempt. The cyclist performed a ‘full‐dress rehearsal’ to gain an understanding of physiological, aerodynamic and biomechanical changes that occur in order to inform potential strategies to optimise performance during the official attempt. The simulation comprised a unique situation where multiple performance determinants could be examined in depth and used to understand variables relating to cycling hour record performance. Furthermore, to gain a better understanding of the factors that contribute to task failure, an even‐pacing strategy was employed where the point of task failure was defined as the lap, which the cyclist could no longer perform at the targeted lap split or quicker; however, once task failure occurred, the participant continued to complete the hour duration. The hypothesis was that there would be significant changes in the parameters when task failure was achieved.

## 2 METHODS

### 2.1 Participant

The participant was a male elite international road and track cyclist (age, 29 years; body mass, 75.2 kg; height, 1.84 m and CP, 353 W) who had competed at the elite track World Championship and medalled at the elite road World Championships (Mixed Team Time Trial). They would be considered as Tier 5: World Class according to the Participant Classification Framework described by McKay et al. (2022)

### 2.2 Informed consent and ethics statement

Prior to the simulation, the cyclist gave their written informed consent and specifically requested that the data generated be made available in the public domain. The data collected were observations on a single day without an intervention or experimental protocol. Ethical approval was obtained from Northumbria University in accordance with the principles of the Declaration of Helsinki, except for registration in a database.

### 2.3 Cycling hour record simulation procedures

The cycling hour record simulation was conducted at an international 250 m standard indoor velodrome (National Cycling Centre) in February 2021, using the cyclist's track bicycle (Electron Pro, Argon 18).

After arriving at the velodrome and conducting the initial logistical preparation, the cyclist's body mass was measured and a capillary blood sample obtained for the measurement of lactate concentration. The cyclist then performed a pre‐determined warm‐up on a stationary trainer (Lemond Fitness, Revolution 1.1), which started approximately 60 min before the start of the attempt. The warm‐up composed of approximately 22 min at 63% of CP, 3 min progressive ramp up to 80% CP and finally, 2 min at predicted hour‐attempt power (∼360 W). After the warm‐up, body mass and rate of perceived exertion (RPE) were measured and another blood sample was obtained. The cyclist then had 16 min to prepare themselves for the simulation. During this time, the race skinsuit and race overshoes (Vorteq Sports, UK), race helmet, (Kask Mistral, Italy) were fitted, along with biomechanical sensors and the HR monitor. All equipment used were within the rules of the UCI for a cycling hour‐record attempt.

Once ready, a short track familiarisation effort on the velodrome was performed. The cyclist was given eight laps to build speed before performing 4 km at their pre‐determined race speed (which took an approximately 9 min). Upon completion of the familiarisation effort, the cyclist was instructed to exit the track. RPE was measured and another blood sample was obtained. Subsequently, his track bike was mounted onto a start gate (Starting Gate, Swiss Timing, Switzerland), which was positioned on the ‘pursuit line’. A further 7 min of passive rest was given before the simulation was started using an international standard count down timer system (Swiss Timing, Switzerland) from 50 s.

Throughout the simulation, feedback was given to the cyclist using four methods, which were within the rules of the UCI for a cycling hour‐record attempt: (1) each lap, the cyclist was informed of the previous lap split. This was done by an investigator verbally shouting the last 2 digits of the lap split. For example, ‘6, 5’ would be a 16.5 s lap; (2) predicted distance, based on the average speed to that point, was given every 5 minutes. This was written on a white board and shown to the participant for three laps to ensure it was recognised; (3) the velodrome lap board (Swiss Timing, Switzerland) was constantly updated each lap, so the cyclist could see the total number of laps accumulated and (4) an electronic tablet (iPad Pro, Apple), with a 60‐min count down timer, was positioned on the track side in full view of the cyclist for the duration simulation.

After precisely 60 min, the track bell was rung to inform the cyclist that the hour was concluded and to complete a further full 250 m lap of the track. The video analysis software (Dartfish, Switzerland) was used to measure the final distance achieved. The total distance covered in the hour was calculated as per the UCI regulation 3.5.031 (UCI Constitution and Regulations, 2023). After dismounting the bicycle at the end of the simulation, body mass was measured and two blood samples were obtained within 2 minutes of completing the effort.

### 2.4 Target distance rationale

The air density of the velodrome, where the actual attempt would take place (Tissot Velodrome), was expected to be approximately 1.130 kg/m^3^. Therefore, prior to performing the simulation, the air density was measured (1.179 kg/m^3^) and the target distance equivalent lap split was adjusted to estimate the equivalent power required. To achieve 54,000 m with an air density of 1.179 kg/m^3^, the cyclist would need to achieve 54,526 m at an air density of 1.130 kg/m^3^, which would break the British record and, unofficially, be second for the unified records at that time. Therefore, his target was a 26.0 s opening lap followed by subsequent 16.6 s laps.

### 2.5 Measurements

*Environmental conditions.* Atmospheric conditions, including ambient temperature, relative humidity, barometric pressure and air density, were measured continuously (0.5 Hz) throughout using a weather metre (Kestrel 5200 Professional Environmental Metre, Kestrel Metres).

*Anthropometry.* Height and body mass were measured using a stadiometer (213 Seca Portable stadiometer) and a weighing scale (Withings Body^+^ Smart Scale, Withings, France), respectively.

*System mass.* The weighing scale was also used to measure system mass, which included everything that was on the bike during the simulation (including bike, clothing, sensors and fluid/food consumed) immediately prior to starting their attempt.

*Critical power (CP).* CP was estimated using training data and targeted efforts in previous 2 months in the preparation for this attempt. Average power outputs were obtained during sustained maximal efforts over 3‐ and 20‐min durations (using the athletes track bike in a TT position on both a stationary trainer and the track). Time and power data were fitted with the inverse linear model; P = *W'* · (1⁄t) + CP.

*Power, cadence and wheel speed.* Instrumented cranks were used to measure power and cadence throughout the warm‐up and attempt (SRM Track Science 8th Generation Power Meter, Schoberer Rad Messtechnik, Germany). The power meter had been recently serviced and calibrated before the simulation effort. A zero offset was performed before the start of the track familiarisation effort. The gear used was 64:14 and wheel speed was measured using a Wahoo Blue SC magnet‐based system (Wahoo Fitness). All parameters were recorded on a cycling computer (Garmin 530, Garmin).

*Heart rate.* HR was collected continuously from the start of the turbo warm‐up until 30 min post simulation via a wireless telemetry system (Wahoo Tickr, Wahoo Fitness) and recorded to the same cycling computer.

*Blood lactate sampling and analysis*. Capillary blood samples (5 μL) were taken from the fingertip and analysed immediately for blood lactate concentration using a handheld analyser (Lactate Pro 2, Arkray), which has a reported overall measurement error of ∼3% (Bonaventura et al., 2015).

*Estimated body temperature.* Estimated body temperature was estimated using a wearable device (CORE, GreenTEG AG) on the HR monitor strap that was sampled every 30 s and recorded to the cycling computer. The wearable device has shown acceptable levels of reliability, albeit with low agreement, during a 60‐min of steady‐state cycling (Verdel et al., 2021).

*Coefficient of aerodynamic drag (C*_*D*_ *A).* C_D_A were calculated using a bicycle‐mounted pitot tube computer (Notio Konect [NK], Notio Technologies) at a frequency of 4 Hz. The NK has previously been shown to be reliable and sensitive (Kordi et al., 2021). The NK was calibrated as per manufactures instructions, using the track familiarisation effort. The NK was also synchronised wirelessly and used to record the power, cadence and wheel speed.

*Kinematic analysis.* Kinematic data were collected with the use of 5, three‐axis gyroscope and three axis accelerometers (Leomo Labs Type‐S Leomo). The sensors were attached to the thigh, foot and inside the tail of the helmet. These sensors have been shown to be a valid and reliable tool for analysing the ranges of motion of the cyclist's lower limbs in the sagittal plane (Plaza‐Bravo et al., 2022). The foot segment range of the first quarter (Q1), that is, from the top dead centre 90° forward from the direction of travel, thigh segment range and pedal smoothness were all calculated. Data were recorded at 4 Hz.

### 2.6 Data and statistical analysis

All data were averaged per minute or per lap, where appropriate, and presented as mean ± SD. Power and speed were also represented as 60 s rolling averages. Once the point of task failure was identified, all data prior to and after this point were averaged and compared using an independent *t*‐test. Significance was accepted at *P* < 0.05.

## 3 RESULTS

The average environmental conditions were ambient temperature, 26.3°C; relative humidity, 23.8%; barometric pressure, 1016.9 mb and air density, 1.179 kg/m^3^, which were relatively constant throughout.

**FIGURE 1:**
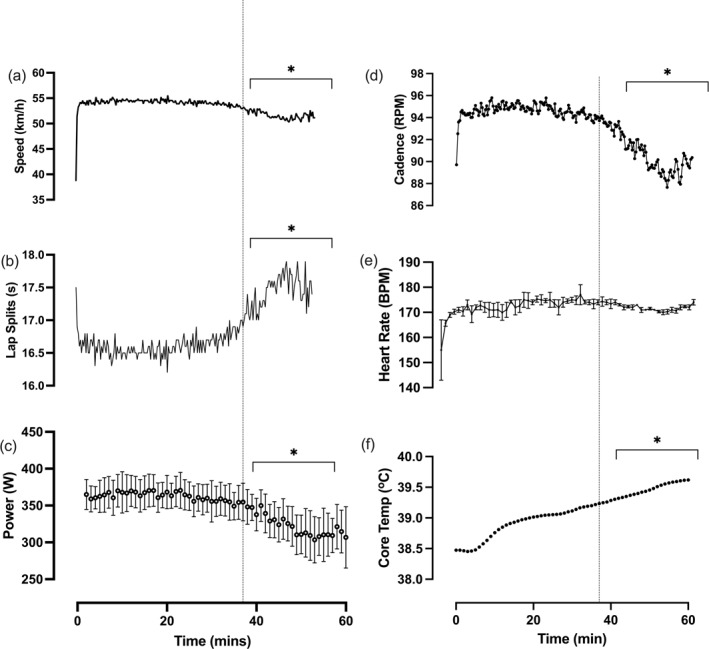
Performance and physiological characteristics throughout the hour record simulation. (A) Speed, (B) lap time, (C) power, (D) cadence, (E) heart rate and (F) estimated body temperature. Dotted vertical line denotes the point of task failure; * denotes a significant difference between post‐task failure compared to pre‐task failure periods. All data are presented as mean ± SD.

The final distance achieved for the hour was 53,250 m. Average speed, lap time, power and cadence were 14.91 ± 0.16 m/s (53.250 km/h^−1^), 16.88 ± 0.60 s, 348 ± 37 W and 93 ± 4 RPM, respectively. Task failure occurred at 38 min 33 s (lap 139/34,750 m). All measures were lower during post‐task failure compared to pre‐task failure periods (Figure 1, Table 1).

**TABLE 1:** Average values of measured parameter pre‐ and post‐task failure. * denotes a significant difference between post‐task failure compared to pre‐task failure periods.

	Pre‐task failure	Post‐task failure	*p*‐value
Speed (m/s)	15.12 ± 0.59	14.52 ± 0.33	<0.001
Lap time split (s)	16.64 ± 0.58	17.33 ± 0.33	<0.001
Power (W)	363 ± 29	320 ± 22	<0.001
Cadence (RPM)	94 ± 4	90 ± 2	<0.001
Heart rate (BPM)	172 ± 4	172 ± 2	0.999
Estimated core body temperature (°C)	38.9 ± 0.3	39.5 ± 0.1	<0.001
C_D_A (m^2^)	0.1635 ± 0.0021	0.1642 ± 0.0035	<0.001
Helmet angle (°)	36.4 ± 1.5	43.2 ± 2.2	<0.001
Helmet rotation (°)	8.0 ± 0.7	10.6 ± 1.3	<0.001
Helmet rock (°)	6.5 ± 0.7	10.0 ± 1.3	<0.001
Foot segment range (°)	46.0 ± 1.6	49.0 ± 2.5	<0.001
Foot segment range (Q1) (°)	20.1 ± 3.8	17.7 ± 4.6	0.031
Thigh segment range (°)	51.8 ± 1.2	54.0 ± 1.4	<0.001
Leg smoothness (%)	8.1 ± 2.1	10.7 ± 3.2	0.153

*Note*: All data are presented as mean ± SD.

The average heart rate was 172 ± 4 BPM. This remained constant between the pre‐ and post‐task failure periods (Figure 1, Table 1). Estimated body temperature increased progressively throughout and was higher during the post‐compared to pre‐task failure periods (Figure 1, Table 1) and had reached 39.2°C at the point of task failure, continuing to increase to 39.6°C at the end.

Blood lactate concentration was 1.3 mmol.L^−1^ at baseline and 1.7 mmol.L^−1^ following the warm‐up. Following the short track familiarisation effort, blood lactate concentration had increased to 3.5 mmol.L^−1^. Immediately following the hour simulation, blood lactate concentration had increased to 11.8 and 12.9 mmol.L^−1^ for the two samples taken, respectively. Body mass decreased slightly from 76.6 kg at baseline to 76.3 kg following the warm‐up and short track familiarisation effort and further declined to 74.8 kg (∼2%) immediately following the simulation.

**FIGURE 2:**
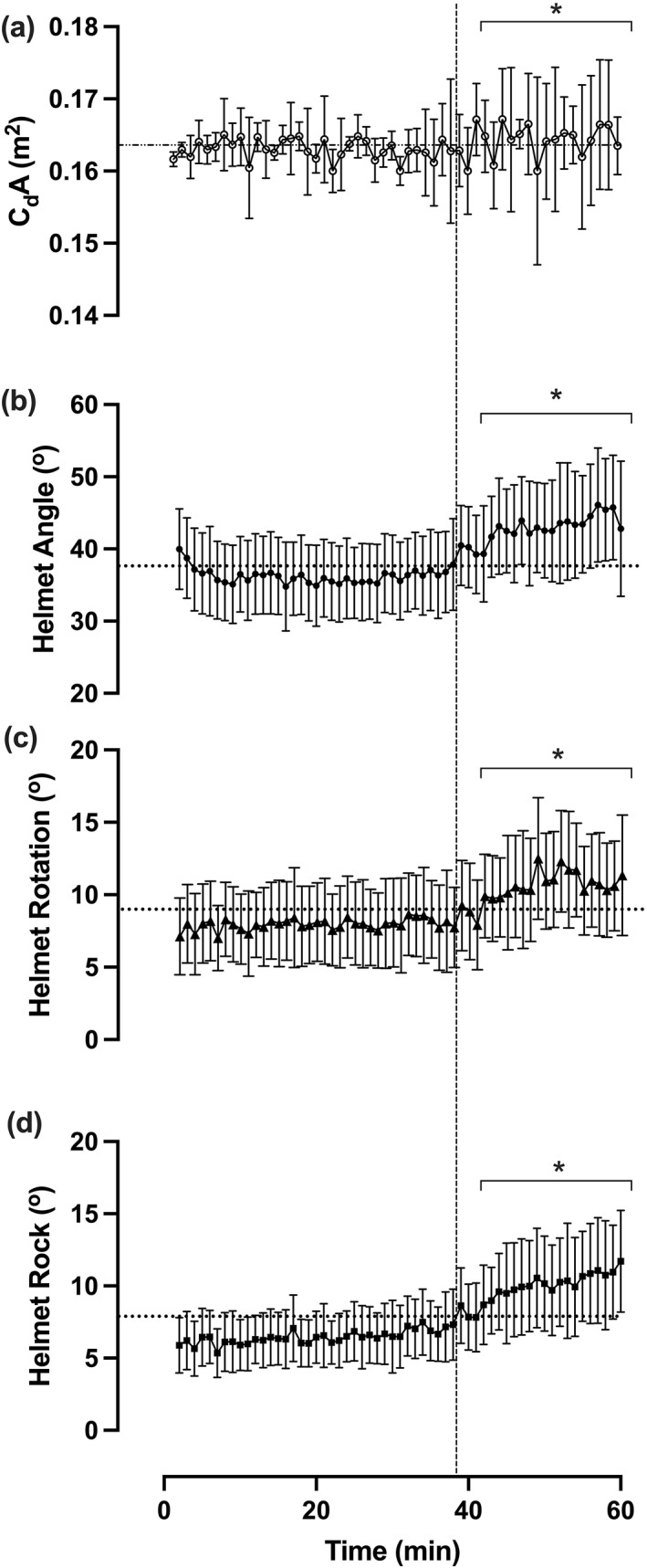
Aerodynamic characteristics throughout the hour record simulation. (A) C_D_A, (B) helmet angle, (C) helmet rotation, (D) helmet rock. Dotted vertical line denotes the point of task failure; * denotes significant difference between post‐task failure compared to pre‐task failure periods. All data are presented as mean ± SD.

**FIGURE 3:**
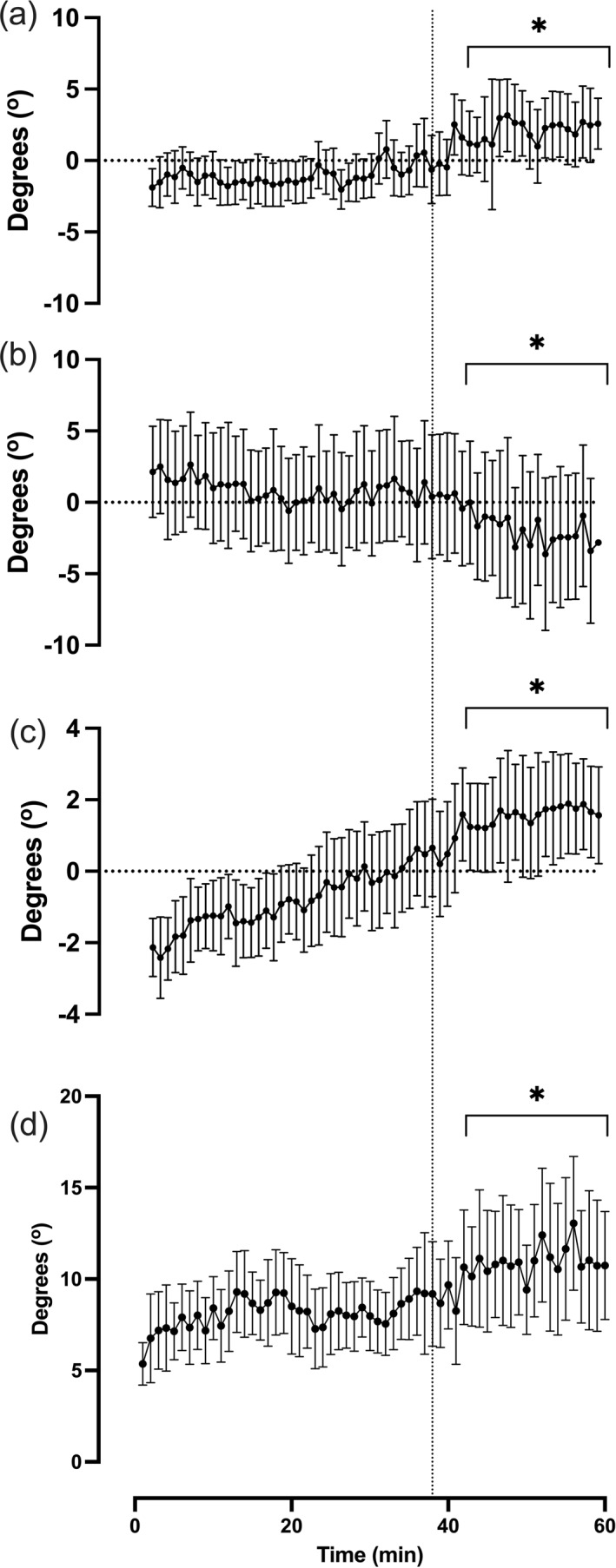
Aerodynamic characteristics throughout the hour record simulation. (A) Foot segment range, (B) foot segment range during the first 90° ([Q1]) of the pedalling cycle, (C) thigh segment range, (D) leg smoothness. Dotted vertical line denotes the point of task failure; * denotes a significant difference between post‐task failure compared to pre‐task failure periods. All data are presented as mean ± SD.

Average C_D_A, helmet angle, rotation and rock throughout were 0.1637 ± 0.0027 m^2^, 38.8 ± 3.7°, 9.0 ± 1.6° and 7.7 ± 1.9°, respectively. All measures were lower during the post‐task failure compared to pre‐task failure periods (Figure 2, Table 1). Foot segment range, foot segment range (Q1), thigh segment range and leg smoothness throughout were 47.1 ± 1.9°, 19.3 ± 4.1°, 52.6 ± 1.3° and 9.0 ± 2.5%, respectively. Foot and thigh segment ranges were higher, foot segment range (Q1) was lower and leg smoothness remained unchanged during the post‐task failure compared to pre‐task failure periods (Figure 3, Table 1).

## 4 DISCUSSION

This case study provides unique insight into physiological, aerodynamic and biomechanical factors over the course of a cycling hour record simulation in an elite cyclist. During the simulation, the cyclist employed an even‐pacing strategy, attempting to maintain a constant lap split (16.6 s) and thus maintain a constant power output (and riding position) to achieve a target distance of 54,000 m. The cyclist was on course to break the target distance (task success) until 38 min 33 s (lap 139/34,750 m) into the simulation, at which point he could no longer maintain the speed required to achieve the target distance (task failure). Task failure corresponded with significant changes in physiological, aerodynamic and biomechanical determinants of hour‐record performance.

Task failure was likely due to multiple physiological fatigue factors resulting in a decrease in power output coupled with a deterioration of optimal aerodynamic positions. The physiological determinants of task failure during an intense exercise are complex and multifactorial (Porter, 1981). Clearly, skeletal muscle fatigue is a major factor, the mechanism of which could be central (e.g., reduction in voluntary activation of the muscles) and/or peripheral (e.g., accumulation/depletion of muscle metabolites) in origin, although, for practical reasons, we were unable to obtain data in relation to these processes. Over the first 30‐min of the simulation (that led to task failure), the cyclist maintained a power output of ∼360 W, which was higher than his estimated CP (353 W). This supports the previous observation that Miguel Indurain's estimated power output during his record‐breaking attempt (∼510 W) was slightly higher than his velodrome estimated onset of blood lactate accumulation threshold of 501 W (Malizia et al., 2021). Regardless of how these important ‘physiological thresholds’ are defined, both case reports indicate that cyclists are above their respective critical threshold and working within the severe intensity exercise domain. Exercise in this domain is strongly associated with a substantial contribution from anaerobic energy pathways resulting in progressive changes in intramuscular substrates and metabolites which increase/decrease until their respective maxima/minima are obtained (Jones et al., 2008; Poole et al., 1988), all of which contribute to the muscle fatigue process (Gandevia, 2001; Sundberg et al., 2019). Indeed, the high blood lactate concentration (∼12 mmol.L^−1^), measured on completion of the simulation, supports this notion. These observations also corroborate with the cyclist's feedback that they were ‘flat out’ and working at a severe intensity.

Task failure, the fatigue process, was undoubtedly influenced by environmental conditions. The ambient temperature of the velodrome was 26.3°C, which, alongside wearing a full skinsuit represents a significant thermal stress. Estimated core body temperature increased from ∼38.5°C at the start to 39.2°C at the point of task failure and continued to increase to 39.6°C at the end. There is strong evidence that a high internal body temperature contributes to fatigue in trained subjects during prolonged exercises, particularly in uncompensable hot environments (González‐Alonso et al., 1999b). The mechanisms are not fully understood, but it is well‐established that adenosine triphosphate utilisation may be increased during exercise in the heat, which is met by an increase in anaerobic glycolysis and muscle glycogen utilisation (Febbraio et al., 1994). This is related to the elevated muscle temperature (Febbraio et al., 1994) and exacerbated if dehydration occurs (González‐Alonso et al., 1999a). The compounding effects of dehydration during exercise in hot environments are also significant, including a reduction in skeletal muscle blood flow to the exercising muscles and reduced muscle O_2_ uptake (González‐Alonso et al., 1998). The extent of dehydration sustained by the cyclist was not assessed directly. However, body mass declined by ∼2%, most of which was likely due to fluid loss through sweat. Moreover, this level of dehydration is well within the normal ranges that are considered to be a major determinant of exercise performance and therefore unlikely to be a factor in task failure (Cheung et al. (2015)/Wall et al. (2015)). Either way, these observations align with the cyclist's reported perceptual feedback, ‘After around 40 min, I was just so unbearably hot. I lost control of my breathing and just couldn't maintain the pace anymore’.

The other major contribution to the reduction in speed was the significant increase in C_D_A. The head and helmet can contribute up to 19% of cyclists C_D_A in the time‐trial position, the most of any body part or segment (Malizia et al., 2021). In this case study, helmet movement significantly increased after task failure. Indeed, narrative feedback provided by the cyclist, suggested they were attempting to deliver more power in order to attenuate any further decreases in speed. This consequently compromised the body position, and the athlete could ‘sense’ that their head was moving more than before, which likely contributed to the increase in C_D_A.

The pedalling kinematics data provide insight that allow us to make some inference on potential changes in the movement strategy leading up to and once task failure is reached. Although these data are limited because the instrumentation only provides segment ranges of motion (i.e., the difference between the minimum and maximum segment angle within a pedal cycle), the observations clearly show that the range of motion of both the thigh and foot segments increased as the task became more challenging. These increases in the segment angle range suggests that under a change in task constraints (reduction in performance), the participant explores alternative movement strategies in order to complete the task goal in a different manner (Martin et al., 2009). It was not possible to say if there was an associated systematic shift in the pedalling motion under fatigue, but this could be the focus of future studies. Nevertheless, the data from this study suggests that there is a change in pedalling kinematics, leading up to and following task failure. However, it is worth noting that previous studies have suggested that changes at the physiological or psychological levels are the cause of reduction in performance and changes in kinematics are the consequence, and are likely to exacerbate task failure (Allen et al., 2008; Gandevia, 2001; Martin et al., 2009).

Only one other case study, published over 20 years ago, has reported observations from test/practice efforts prior to a successful cycling hour record attempt that exceeded 53,000 m (Padilla et al., 2000). The authors reported wind tunnel C_D_A values of 0.244 m^2^ and, making assumptions of the atmospheric conditions which were not reported, suggested that the average power that was required to achieve the target distance was ∼510 W. The current case study suggests that the cyclist produced less power (38%) and had a lower C_D_A (39%; although we acknowledge that C_D_A calculated from testing in a velodrome cannot be directly interchanged with wind tunnel tests (Kordi et al., 2021)). These observations are perhaps not surprising given the significant advancements in aerodynamic technology over the last 20 years, including bicycle design, helmet and skinsuit material and design, as well as optimal on‐bike‐positioning achieved through wind tunnel testing.

### 4.1 Practical applications

The present case study suggests that the reduction in performance (leading to task failure) during the cycling hour record is underpinned by a decrease in power output as well as an increase in aerodynamic drag. Given the significant thermal stress encountered that likely contributed to task failure, methods that can attenuate the increase in body temperature should be employed in the preparation of the athlete (e.g., heat acclimation and pre‐event cooling strategies). It is noteworthy that some of these interventions were performed in the lead up to his subsequent successful attempts where he went on to break the British hour record within 6 months and the official UCI cycling hour record a year later. Moreover, a more conservative ‘negative split’ pacing strategy, rather than sustained even‐pace from the start, was used in the subsequent successful attempt (achieving 55,548 m).

### 4.2 Limitations

The aim of this case study is to focus on the performance of the hour record and therefore is limited by the lack of detailed insight into the preparatory training and the cyclist's nutritional strategy in the weeks and days leading up to the attempt. Moreover, we did not provide any further insights into the psychological determinants of cycling hour record performance. Indeed, the decision to partially disengage (i.e., reduce effort) or terminate endurance exercise (task failure) is impacted by both psychological and physiological factors (Marcora et al., 2009). In such situations, motivational dynamics may play a critical role in overall exercise performance whereby the desire to reduce effort during exercise conflicts with the performance goal of the task (Taylor et al., 2022).

## 5 CONCLUSION

This case study provides a rare insight into the limits of human performance during a cycling hour record simulation in an elite cyclist. It provides a unique understanding of physiological, aerodynamic and biomechanical changes throughout the task. From this, it seems that both propulsive (i.e., mechanical power output) and resistive forces (i.e., C_D_A) are significantly altered, both of which have a major influence on task outcomes (success or failure). These data could provide the catalyst for others examining the limits of human performance during arduous endurance tasks.

## CONFLICT OF INTEREST STATEMENT

The authors declare that they have no conflicts of interest.
